# A Non-Specific Effect Associated with Conditional Transgene Expression Based on Cre-loxP Strategy in Mice

**DOI:** 10.1371/journal.pone.0018778

**Published:** 2011-05-10

**Authors:** Linghua Qiu, Jaime A. Rivera-Pérez, Zuoshang Xu

**Affiliations:** 1 Department of Biochemistry and Molecular Pharmacology, University of Massachusetts Medical School, Worcester, Massachusetts, United States of America; 2 Department of Cell Biology, University of Massachusetts Medical School, Worcester, Massachusetts, United States of America; 3 Neuroscience Program, University of Massachusetts Medical School, Worcester, Massachusetts, United States of America; Seattle Children's Research Institute, United States of America

## Abstract

Transgenes flanked by loxP sites have been widely used to generate transgenic mice where the transgene expression can be controlled spatially and temporally by Cre recombinase. Data from this approach has led to important conclusions in cancer, neurodevelopment and neurodegeneration. Using this approach to conditionally express micro RNAs (miRNAs) in mice, we found that Cre-mediated recombination in neural progenitor cells caused microcephaly in five of our ten independent transgenic lines. This effect was not associated with the types or the quantity of miRNAs being expressed, nor was it associated with specific target knockdown. Rather, it was correlated with the presence of multiple tandem transgene copies and inverted (head-to-head or tail-to-tail) transgene repeats. The presence of these inverted repeats caused a high level of cell death in the ventricular zone of the embryonic brain, where Cre was expressed. Therefore, results from this Cre-loxP approach to generate inducible transgenic alleles must be interpreted with caution and conclusions drawn in previous reports may need reexamination.

## Introduction

The Cre-loxP system has been used widely for conditional transgene expression in mice. A common approach is to make transgenic mice by pronuclear injection of transgene constructs where the transgene is flanked with loxP sites. This approach has been adopted for either conditional induction or cessation of transgene expression in a spatially and temporally controllable manner by crossing with various Cre-expressing driver lines. Since its first demonstration in mice in 1992 [1], [2], this approach has been used to conditionally express toxic genes for cell ablation [3], [4], [5], short hairpin RNAs (shRNAs) for gene silencing [6], [7], disease-associated genes for defining their roles in neurodegenerative disorders [8], [9], [10], [11], [12] and marker proteins for labeling different cell populations in the central nervous system (CNS) [13], [14]. To take advantage of this powerful approach, we applied it for conditional expression of several miRNAs targeting specific genes in mice.

## Results

**Figure 1:**
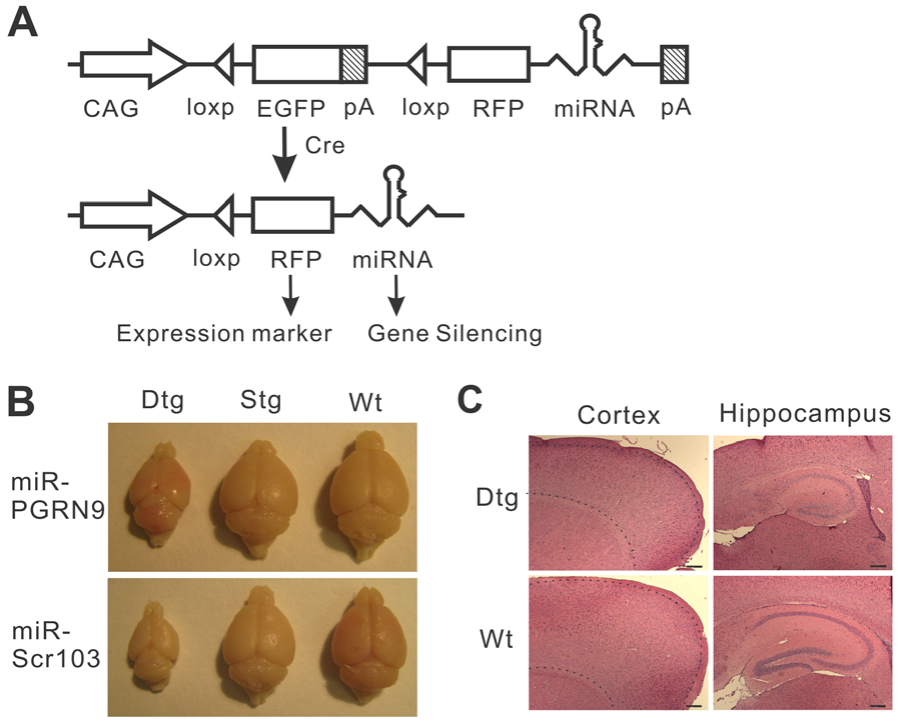
Cre-loxP-based conditional miRNA expression causes microcephaly. (A) Schematic diagram of the conditional miRNA expression strategy. This construct initially expresses EGFP driven by the CAG promoter. After Cre-mediated DNA recombination between two loxP sites, the loxP-flanked EGFP gene is excised, allowing the promoter to drive the expression of the marker RFP and the miRNA that silences its target gene. pA stands for polyA signal. (B) Brains from the miRNA and nestin-Cre double transgenic mice (Dtg) are smaller than those of the single transgenic (Stg) or wild type (Wt) littermates. (C) Hematoxylin and eosin (H&E) stained sagittal sections from of the brains of 21-day-old Dtg (miR-E1k32/nestin-Cre) and Wt mice. The boundaries of the cortex are marked by dotted lines. Scale bar = 200 µm.

We used the previously characterized construct pCAG-EGFP/RFP-miRNAint (G/R-miRNA) to express miRNAs in transgenic mice (Fig. 1A) [15]. This construct first expresses enhance green fluorescent protein (EGFP), which enables rapid screen of transgenic mouse lines where the transgene is active in the desired tissue. Upon induction by Cre, the EGFP gene is excised, leaving the promoter to drive the expression of red fluorescent protein (RFP) and miRNA. The RFP provides a convenient indicator for the level and location of the miRNA expression [15]. We targeted two genes with this construct. One was the progranulin gene and the other was the E1k subunit of á-ketoglutarate dehydrogenase complex (KGDHC). Both genes are involved in neurodegenerative diseases. Loss of function mutations in the progranulin gene cause frontotemporal dementia (FTD) and a decrease in KGDHC activity is associated with Alzheimer's disease (AD) [16], [17], [18]. As a control for possible non-specific effects associated with the overexpression of miRNA, we used a construct that expresses a scrambled miRNA (miR-Scr) that does not target any specific gene.

Transgenic mice were generated by pronuclear injection. Two constructs for progranulin knockdown (miR-PGRN1 and 9), one construct for E1k knockdown (miR-E1k) and one construct with scrambled miRNA sequence (miR-Scr) were injected. Transgenic lines were screened based on a semi-quantitative estimation of EGFP fluorescence in the brain. One transgenic line from each of the two miR-PGRN constructs, two lines from the miR-E1k and six lines from the miR-Scr were selected and propagated based on their relatively high levels of EGFP expression in the brain (Table 1). The transgenes were inherited at a frequency expected from Mendelian inheritance without any overt phenotypes. To investigate the effect of PGRN and E1k knockdown in the brain, we crossed the offspring of these lines with nestin-Cre driver mice, which express Cre in neural progenitor and glial cells beginning at ∼E10.5 day [19]. In half of the transgenic lines, the double transgenic progeny developed microcephaly. The brains of these double transgenic mice (Dtg) weighed approximately 50% of those of their single transgenic (Stg) and wild type (Wt) littermates (Fig. 1B; Table 1). The overall brain structures were maintained but were proportionally smaller than those of the Stg and Wt littermates. For example, the cortical cell layers appeared normal but thinner (Fig. 1C). Similarly, the hippocampal architecture appeared intact but smaller compared with the non-transgenic brains (Fig. 1D). The microcephaly phenotype was observable at birth. However, in most lines the number of the double transgenic progeny was not different from what was expected from Mendelian inheritance (Table 2), indicating that the microcephaly did not cause embryonic lethality in most lines.

**Table 1:** Summary of basic characteristics of the ten transgenic lines.

Tg line	G/R-miR-PGRN		G/R-miR-E1k		G/R-miR-Scr					
	9	1	32	15	103	35	97	105	109	113
GFP (Stg)	++++	+++	++++	++++	+++	++++	++++	++++	+++++	+++++
Br wgt (Dtg/Wt)	0.44	0.98	0.55	0.97	0.41	0.51	0.54	0.93	1.01	1.1
RFP (Dtg)	+	++	−	+++	−	++	+	+++	+++	+++
mRNA (Stg%Wt)	86±16	96±7.6	101±3.5	91±10	NA	NA	NA	NA	NA	NA
mRNA (Dtg%Wt)	76±4.7	100±6.3	78.5±6.2	23±10	NA	NA	NA	NA	NA	NA
miRNA (Stg/Wt)	742	301	189	510	948	ND	3295	ND	6838	5082
miRNA (Dtg/Wt)	2381	568	92	1960	130	ND	2272	ND	8352	6803
Tg copy #	28.4±7.6	0.7±0.1	9.4±1.8	2.3±0.1	21.1±5.2	1.6±0.3	15.9±5.7	1.5±0.1	1.5±0.1	1.3±0.1
H-H junction	+*	−	+	−	+	+*	−	−	−	−
T-T junction	+*	−	−	−	+*	−	+	−	−	−
H-T junction	+	−	+*	+*	+*	−	+*	−	+*	−

**Figure 2:**
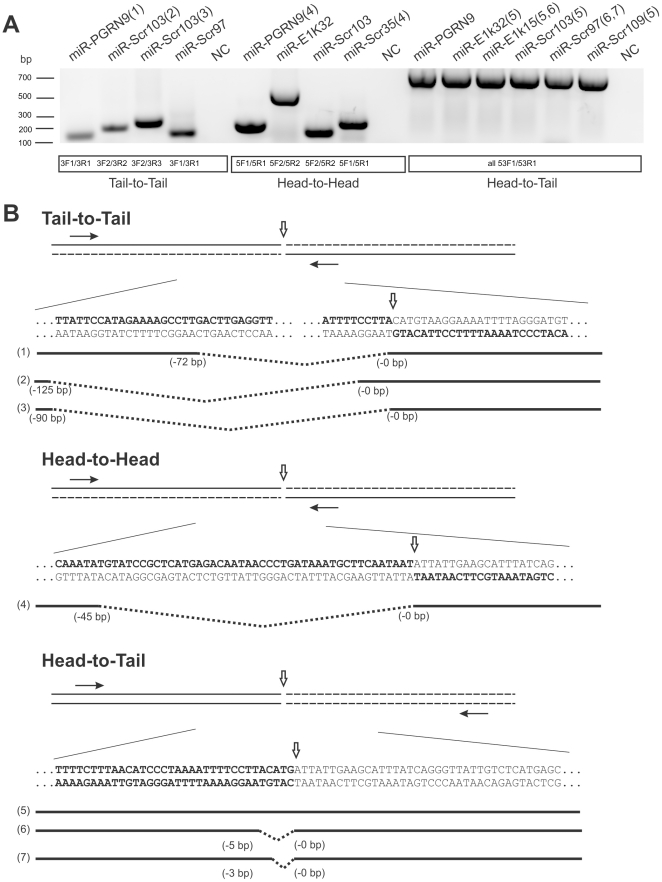
Detection of inverted transgene arrays in transgenic lines. (A) PCR products of tail-to-tail, head-to-head and head-to-tail junctions in seven out of ten transgenic lines (see Table 1). DNA samples from different transgenic mouse lines are shown in each lane. The positions of the primers are indicated in (B) and their sequences are shown in Table 3. (B) Sequences were successfully read from some of the PCR bands shown in (A) and these bands are marked with numbers in the parentheses. Some bands contain two sequences that can be read and these are marked by two numbers in the parentheses. The bands without sequences represent those that could not be read, most likely due to multiple sequences in the band. NC is a control in the PCR reaction using the DNA from a non-transgenic mouse. Sequences obtained from the numbered bands are aligned with the expected junctional sequences from the construct. With only one exception (the #5 band), all other junctional bands had deletions, which are marked by dotted lines between the solid lines. The solid lines represent the detected sequences. The position of the junctions is marked with negative numbers for the fragment on the left and positive numbers for the fragment on the right.

The GFP and RFP fluorescence intensity in the brain was examined under the fluorescent microscope and different intensities of the fluorescence were graded from the weakest “+” to the strongest “+++++”. The brain weights are expressed as ratios of the average from double transgenic mice to that of the age-matched wild type mice. The average weights with standard deviations and the statistics are shown in Table S1. The levels of mRNA and miRNA were determined by real-time RT-PCR. The mRNA levels of the double transgenic mice are presented as a percentage of the wild type mice. The miRNA levels of the double transgenic mice are presented as fold increases over the background value obtained from wild typemice. The transgene copy numbers were determined by real-time PCR of the genomic DNA from single transgenic miRNA lines. The different types of junctional fragments were detected by PCR using specific primer pairs (see Table 3). Stg: single transgenic mice positive for the G/R-miRNA transgene only.Dtg: double transgenic mice positive for both the G/R-miRNA transgene and nestin-Cre. Wt: non-transgenic mice. Br: brain. Wgt: weight ratio of Dtg-to-Wt. Stg%Wt: level in Stg as a percent of Wt. Dtg%Wt: level in Dtg as a percent of Wt. Tg: transgenic. H-T: head-to-tail.H-H: head-to-head. T-T:tail-to-tail. “+” indicates positive for the specific junctional PCR product. “−” indicates negative for the specific junctional PCR product. “+*” indicates that this PCR product sequence was successfully read (see Fig. 2).

**Table 2:** Genotype composition of the progenies from the crossings of G/R-miRNA and nestin-cre.

Tg Line	miR-PGRN9	miR-E1k32	miR-Scr103	miR-Scr35	miR-Scr113
Dtg mice	13	3	13	19	18
Stg mice	44	4	11	18	14
Wt	43	11	13	22	12
nestin-Cre	39	7	10	25	15
total	139	25	47	84	59
P value	0.003	0.856	0.464	0.867	0.957

Genotype composition of the progenies from the crossings of G/R-miRNA and nestin-Cre. The number of the various genotypes was compared with the Mendelian ratio using the Chi Square test to obtain the P value.

The microcephaly might be caused by the knockdown of PGRN or E1k expression, but this appeared unlikely. In the miR-PGRN9 line which had microcephaly, there was only ∼25% knockdown of PGRN mRNA (Table 1). This is unlikely to cause microcephaly because this phenotype was not observed in the progranulin knockout mice [20], [21], [22], [23]. In the miR-E1k lines, line 32 developed microcephaly but line 15 did not. However, line 32 had only ∼20% knockdown of the E1k mRNA whereas line 15 had ∼75% knockdown. Thus, target knockdown could not explain the cause of microcephaly.

It has been shown that high levels of siRNA or shRNA expression can cause toxicity in mice [24], [25]. Therefore, high levels of miRNA expression might also be toxic and cause microcephaly. To test this possibility, we first analyzed the RFP expression in the double transgenic progeny since the RFP expression was driven by the same promoter that drove expression of the miRNA (Fig. 1A). We found that the levels of RFP did not correlate with the microcephaly phenotype. For example, the transgenic lines with the highest RFP levels, such as miR-E1k15, miR-Scr105, miR-Scr109 and miR-Scr113, did not develop microcephaly, whereas the lines with low levels of RFP expression, such as miR-E1k32 and miR-Scr103, developed microcephaly (Table 1). To verify the miRNA levels, we measured the transgene-encoded miRNAs by real time RT-PCR and confirmed that most transgenic lines with the highest miRNA levels did not develop microcephaly, whereas the lines with relatively low levels of miRNA did (Table 1). Some transgenic lines also had a significant level of leakage in miRNA expression before crossing with nestin-Cre (Table 1), but none showed microcephaly during line propagation in crossings with the wild type mice. Thus, the microcephaly phenotype did not correlate with high levels of miRNA expression.

To further seek the cause for this phenotype, we measured the transgene copy numbers in all the transgenic lines using real time PCR of the genomic DNA. Interestingly, four out of five transgenic lines with microcephaly phenotype had high transgene copy numbers, while all lines without microcephaly had low transgene copy numbers (Table 1). This observation raised the possibility that aberrant Cre-mediated recombination at the site of the multiple transgene integration might cause the microcephaly. One possible scenario is that a cellular toxicity might be derived from the presence of inverted loxP sites, i.e. loxP sites in a head-to-head or tail-to-tail configuration. For example, transgenes with inverted loxP repeats can cause chromosome loss and death of proliferating cells during Cre-mediated recombination [26], [27]. However, the prevalent assumption has been that multiple transgene copies are integrated in a tandem head-to-tail array with only rare exceptions of inverted repeats [28], [29], [30].

To test the hypothesis that Cre-mediated recombination of the loxP-containing transgenes that are integrated in an inverted manner leads to microcephaly, we used PCR to amplify all possible types of transgene-transgene junctions, including the head-to-tail and the inverted head-to-head and tail-to-tail junctions. We detected head-to-tail PCR products from six out of ten transgenic lines (Fig. 2; Table 1). Four of these six lines (miR-PGRN9, miR-E1k32, miR-Scr103 and 97) had 9 or more transgene copies and also carried inverted transgene integration. The other two lines (miR-E1k15 and miR-Scr109) had only head-to-tail integration and carried 2–3 transgene copies. One line miR-Scr35 carried two transgene copies that are integrated head-to-head. Three lines (miR-PGRN1, miR-Scr105 and 113) had low transgene copies and no junctional PCR products. These lines were likely to carry a single copy of the transgene. To ascertain that the PCR products are truly derived from the expected junctions, we sequenced the junctional PCR products and were able to obtain sequences from most of these fragments (Fig. 2; Table 1). Because of the frequent deletion of the sequences from the original constructs at the junctions, we often observed multiple sequences from one PCR band (Fig. 2). We were able to read sequences from some of the PCR bands that contained two sequences, but were unable to read sequences from several other bands. This was most likely due to the presence of multiple sequences within those bands.

**Table 3:** PCR primers for detection of transgene junctions.

Junction type	Primer	Primer sequence
Head-to-tail	XF1	5′-CAGAAGGTGGTGGCTGGTGTG-3′
	XR1	5′-TGGCGTTACTATGGGAACATACGT-3′
Head-to-head	5F1	5′-(ACCGCCA)CGTAAGTTATGTAACGCGGAAC-3′
	5R1	5′-(GCGAGTGTCGTG)GACAATAACCCTGATAAATGCTTCAATA-3′
	5R2	5′-(CAACGCCAC)CTCATGAGACAATAACCCTGATAAATG-3′
Tail-to-tail	3F1	5′-(ACGAATAGCCGA)CTGGCTGCCATGAACAAAG-3′
	3F2	5′-(GTCCTCCGACA)TCACTCGGAAGGACATATGG-3′
	3R1	5′-(AGCCCAGTGCCC)TTTTTTCTTTAACATCCCTAAAATTTTCCTTA-3′
	3R2	5′-(CGCAGTGCCC)GTTATTTTTTTCTTTAACATCCCTAAAATTTTCC-3′
	3R2	5′-(CGAGTGCCG)GTTTTGTGTTATTTTTTTCTTTAACATCCCTA-3′

Sequences in the parentheses were intentionally designed not to match with the transgene. See Materials and Methods for details.

Notably, all the lines that were positive for the inverted transgene integration, including miR-pgrn9, -miR-E1k32, miR-Scr103, miR-Scr97 and miR-Scr35, had the microcephaly phenotype. In contrast, all the lines that were negative for the inverted transgene integration did not have the microcephaly phenotype. Thus, the presence of the inverted transgene integration predicted the microcephaly. Additionally, of the seven lines that had two or more copies of the transgene, five had inverted transgene integration. The two lines that did not have inverted transgenes had low transgene copies. Thus, contrary to the prevailing assumption that the transgenes are integrated into the genome in a head-to-tail tandem array with rare exceptions [28], [29], [30], the inverted transgene integration is common in mice that have multiple transgene copies.

**Figure 3:**
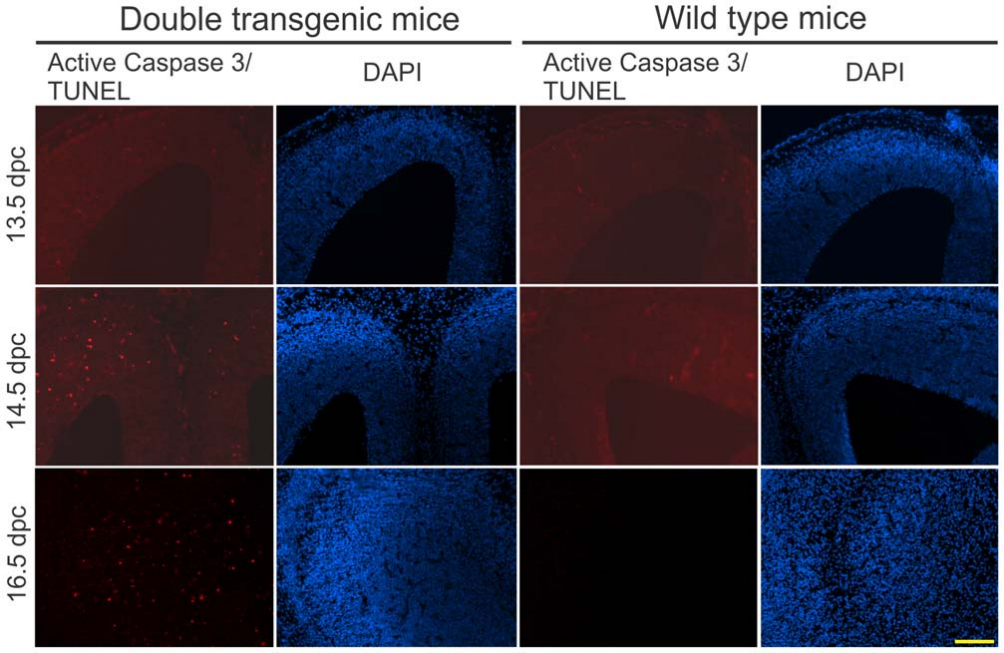
Increased apoptosis in the proliferating cell population in prenatal mouse brains. Embryos of the double transgenic and wild type mice at different stages from line miR-PGRN9 were fixed and frozen brain sections were cut 10 µm thick. The sections from 13.5 dpc (days post coitum) and 14.5 dpc were stained for activated caspase 3 and the sections from 16.5 dpc were stained with TUNEL. The positive signal for apoptosis was mostly observed around the ventricular zone in the double transgenic mice, as shown in the figure. Scale bar = 200 µm.

Finally, to determine whether the inverted transgene repeats caused cell death in proliferating cells, as has been shown previously [27], we examined the double transgenic embryos with both the G/R-miRNA and nestin-Cre transgene. We detected increased cell death in the ventricular zone and the cortex during the neurogenesis at 14.5 and 16.5 days post coitum (dpc) (Fig. 3). This observation confirms that the inverted transgene repeats containing loxP sites caused death of the proliferating cells during neurogenesis, which led to the microcephaly.

## Discussion

While applying the Cre-loxP system for conditional expression of miRNA in mice, we found a common phenotype of microcephaly after Cre-mediated recombination in the developing brain. This phenotype was not correlated with the target gene knockdown or the levels of miRNA expression. Rather it was correlated with the integration of inverted transgene copies in the genome. Examination of mouse embryos showed that Cre-mediated recombination in the developing brain caused an increase in cell death. Based on these lines of evidence, together with previous studies demonstrating that Cre-mediated recombination in cells with inverted loxP sites in the genome can cause chromosome loss and death of proliferating cells [26], [27], we conclude that integration of inverted transgene repeats into the genome is common in transgenic mice made from pronuclear injection. If the transgene contains loxP sites, the inversion can cause death of proliferating cells during Cre-mediated transgene recombination. In the developing brain, this leads to microcephaly.

Our finding explains some of the previous observations in the literature. For example, Lobe and colleagues reported construction of Cre reporter mice using the construct Z/AP [31], which we modified in the current study to generate the G/R-miRNA construct. In the Z/AP construct, the CAG promoter drives the expression of LacZ gene that is flanked by loxP sites. Upon Cre-mediated recombination, the LacZ gene is removed and the CAG promoter drives the expression of the alkaline phosphatase gene. Among the Z/AP transgenic lines that were crossed with CMV-Cre driver (a ubiquitous driver), they obtained the expected induction pattern in a line with a single copy of the transgene. However, they could not obtain any double transgenic offspring in a line with multiple transgene copies. In a later report where another Cre reporter transgenic line was generated, the authors screened for and used only ES cell clones with a single transgene copy for generation of the mice [32]. Based on our findings, it is likely that the transgenic line with multiple transgene copies had inverted transgene integration, which caused death of double transgenic embryos.

It has been well-established that inverted loxP sites can cause chromosome deletion during Cre-mediated recombination in dividing cells. Lewandoski and Martin first described a transgenic line that had an inverted loxP-containing transgene on the Y chromosome. Cre-mediated recombination led to the loss of the Y chromosome [26]. Later studies took advantage of this phenomenon and targeted the inverted loxP sites to specific chromosomes for induction of chromosome deletion as well as various other chromosome rearrangements [33], [34], [35], [36]. Furthermore, it has been shown that Cre-mediated recombination of inverted loxP sites on autosomes can cause cell death in proliferating cells, although non-proliferating cells such as neurons are spared [27], [36].

Although the presence of inverted transgenes was found in mice generated by pronuclear injection in 1980s, it has been thought to occur rarely [28], [29], [30]. This has become the basic assumption since the Cre-loxP technology was first applied to conditionally express transgenes in mice in 1992 [1], [2]. In recent years, the use of loxP-containing transgenes in transgenic mice has increased but the possibility of unintended effects of this approach has not been taken into consideration [5], [7], [8], [9], [10], [11], [12], [13], [14], [37], [38], [39], [40], [41]. Our data demonstrate that, contrary to the widely held assumption that transgenes are integrated into the genome in a head-to-tail tandem array [5], [7], [8], [9], [10], [11], [12], [13], [14], [28], [29], [30], [37], [38], [39], [40], [41], inverted transgene integration is common when multiple copies of the transgene are integrated into the genome. Therefore, the non-specific effects of the inverted loxP sites have been overlooked and can provide alternative interpretations to the previous studies using this conditional transgene expression strategy. For example, a mutant SOD1 transgene that was flanked by loxP sites was used to construct a model for amyotrophic lateral sclerosis (ALS), which was crossed with a P0-Cre driver line. It was intended to delete the mutant SOD1 gene from the Schwann cells, thereby diminishing the expression of the mutant SOD1 in this cell type. The investigators observed that the ALS phenotype was exacerbated and concluded that the SOD1 enzyme activity carried by the mutant protein was protective to motor neurons and the reduction of this activity was responsible for the observed result [10]. Based on our result, an alternative interpretation is that ablation of proliferating Schwann cells during development or during axonal degeneration caused the observed phenotype. In another case, the Cre-loxP strategy was used to conditionally express shRNAs that silenced the fibroblast growth factor receptor 2 (*Fgfr2*) gene. When the shRNA was ubiquitously induced by EIIa-Cre driver, embryonic lethality was observed. When the shRNA was induced in the limb bud of the mouse embryos, an enhanced cell death and malformed limbs developed. The authors attributed these effects to specific silencing of the *Fgfr2* gene [7]. Based on our data, an alternative interpretation is that the non-specific effects associated with the Cre-loxP approach caused the observed phenotypes. In conclusion, it is imperative that investigators who use transgenic lines constructed with loxP-containing transgenes take the non-specific effect into consideration in the experimental design and the interpretation of the results.

## Materials and Methods

### Transgene constructs

A previously described pCAG-EGFP/RFP-miRNAint (G/R-miRNA) construct [15] was used to construct the miRNA expression transgenes (Fig. 1A). The pCAG-EGFP/RFP-miR-E1k (miR-E1-1 in [15]) synthesizes a miRNA that targets the E1k subunit of á–ketoglutarate dehydrogenase complex (KGDHC). The pCAG-EGFP/RFP-miR-Scr synthesizes a scrambled miRNA that targets no specific gene. These constructs were built as described previously [15]. pCAG-EGFP/RFP-miR-Pgrn1 and 9 target the mouse progranulin gene and they were constructed as follows: miRNAs were designed and inserted into the pCAG-EGFP/RFP-miRNAint backbone as described previously [15]. The target sequence of miR-Pgrn1 is 5′-GGACACAUGGCCUAGAAUAACGA-3′. The target sequence of miR-Pgrn9 is 5′- TCCAGAGTAAGTGCCTATCCAA-3′. All constructs were verified by DNA sequencing. The constructs were tested for knockdown efficiency in mouse NF-1 cells as described previously [15] before being used for making transgenic mice.

### Generation and propagation of transgenic mouse lines

The pCAG-EGFP/RFP-miRNAint constructs were linearized by digestion with the restriction enzymes SspI and PciI. The 6.3 kb DNA fragment was isolated from agarose gel, further purified using QIAquick Gel Extraction Kit (Qiagen) and injected into C57BL/6× SJL F2 hybrid mouse embryos by the Transgenic Animal Modeling Core. All mice were maintained at the University of Massachusetts Medical School animal facility according to the guidelines set forth by the Institutional Animal Care and Use Committee (IACUC) and all animal procedures were approved by the IACUC (protocol A1044). Transgenic mice were identified by a 365 bp fragment produced by PCR of genomic DNA extracted from tails or ear clips using the primer pair 5′-AGTGGGAGCGCGTGATGAACTTCGA-3′ and 5′-CTGCTCCACGATGGTGTAGTCCTCGT-3′. The PCR program is 2 minutes at 95°C, followed by 35 cycles of 95°C for 30 seconds, 65°C for 30 seconds, and 72°C for 30 seconds. The transgenic mice were maintained as heterozygotes by crossing to C57BL/6J mice.

To induce the miRNA expression in the central nervous system (CNS), the conditional G/R-miRNA mice were crossed with Nestin-Cre mice (stock #003771, Jackson Laboratory, Bar Harbor, Maine, USA) to obtain the double transgenic mice (G/R-miR;Nes-Cre). Analyses on the double transgenic mice were carried out by comparing them with the wild type and single transgenic littermates. For analysis of mouse embryos, timed matings were carried out and the embryos were obtained from the pregnant females at specified times after they were sacrificed by isoflurane overdose.

### Determination of transgene copy numbers and junctions

Transgene copy numbers were determined by quantitative PCR. Genomic DNAs were extracted and purified from mouse tails by DNeasy Blood & Tissue Kit (Qiagen) according to the manufacturer's instructions. To generate a standard curve, various amounts of pCAG-EGFP/RFP-miRNAint plasmid DNA were added to 396 pg of mouse genomic DNA (equivalent to 60 copies of diploid DNA) from non-transgenic mouse tails at the ratios of 0, 1, 4, 16 and 64 copies per diploid genome. Real time PCR was conducted on duplicates of this standard series and DNA samples (396 pg each) from the transgenic lines using a primer pair 5′-CCGTGAAGCTGAAGGTGAC-3′ and 5′-ATCACGCGCTCCCACTTG-3′, which amplifies a 150 bp fragment of the RFP gene. For each reaction, an internal reference reaction was also conducted using the primer pair 5′-CTCTCTCCGTTGCAGGCAT-3′ and 5′-GTCTTACAGTGAGCTCCGTTC-3, which amplifies a 195 bp fragment of the Fabpi gene. Quantitative PCR was performed using IQ Sybr Green mix (Bio-Rad) according to the manufacturer's instructions. Program for PCR was set as: 95°C for 3 minutes followed by 40 cycles of 95°C for 15 seconds and 57°C for 60 seconds. Melting curves were checked to ensure a single peak. PCR products were run on a 2% agarose gel to verify the DNA size. The Ct values were obtained from the PCR data with the threshold set near the middle of the linear range on a log scale. For each reaction, the ΔCt was calculated by subtracting the Ct value of the reference gene Fabpi from the Ct value of the transgene. The average ΔCt values were plotted against log (transgene copy number) to generate a standard curve. The transgene copy numbers were then derived from the standard curve with the ΔCt values of the transgenic DNA samples.

To determine the transgene junctions, tail DNA samples were prepared as described above. Primers were designed to detect three types of junctions: tail-to-tail, head-to-head and head-to-tail (Table 3). A regular PCR program was used for detection of the head-to-tail junctions: 4 minutes at 95°C, followed by 35 cycles of 30 seconds at 95°C, 30 seconds at 60°C, and 30 seconds at 72°C. A special PCR assay had to be implemented to detect the inverted transgene junctions (head-to-head and tail-to-tail) because the initial regular PCR assay failed to detect these junctions, which was likely due to the potential for folding into hairpin structures by these junctions during the PCR reaction. First, our primer pairs were designed to form asymmetric PCR products that minimize the folding potential for hairpins. For each primer pair, one targeted the sequence near the junction while the other targeted sequence farther from the junction. Second, sequences that were not complementary to the transgene sequence were added to the 5′ end of each primer (Table 3, sequences in parentheses). Third, a two-stage PCR program was implemented. The first stage was 4 minutes at 95°C, followed by 4 cycles of 30 seconds at 95°C, 30 seconds at 51°C and 30 seconds at 72°C. This was followed by the second stage: an additional 40 cycles of 30 seconds at 95°C, 30 seconds at 61°C, and 30 seconds at 72°C. The PCR products were checked on a 2% agarose gel. The DNA bands were purified with QIAquick Gel Extraction Kit (Qiagen) and sequenced to verify that the fragments were derived from the transgene junctions.

### Quantification of the levels of mRNA and miRNA

Total RNA was extracted from mouse tissues by Trizol reagent (Invitrogen) and treated with DNase I (Promega). For mRNA quantification, cDNA was synthesized using SuperScript III kit (Invitrogen) following the manufacturer's protocol. Quantitative PCR was performed by using IQ Sybr Green mix (Bio-Rad). The primer pair 5′- CTAAGGTTGGGAATGTGGAGT-3′ and 5′- AAACATGTCCCAGCGAGGA-3′ were designed for the mouse progranulin gene with a product size of 209 bp. The primer pair 5′-TTCACTCGGGTGGAGCTG-3′ and 5′-ATGAAACATTTTGTCCTGAAGC-3′ were designed for the mouse E1k gene with a product size of 65 bp. Mouse gene glyceraldehyde-3-phosphate dehydrogenase (Gapdh) was used as an internal reference and was amplified with the primer pair 5′-CTGGAGAAACCTGCCAAGTA-3′ and 5′-TGTTGCTGTAGCCGTATTCA-3′, which produced a PCR product of 223 bp. The PCR program was set as follows: 3 minutes at 95°C, followed by 40 cycles of 15 seconds at 95°C, 30 seconds at 56°C, and 20 seconds at 72°C. Ct values were derived from the PCR data with the threshold set near the middle of the linear range on a log scale. ΔCt was calculated by subtracting the Ct value of Gapdh from that of Pgrn or E1k. ΔΔCt values were derived by subtracting the ΔCt values of the wild type from those of the single transgenic and double transgenic mice, which were then used to calculate the levels of the mRNA in the double transgenic mice as a percentage of the level in the wild type mice.

For miRNA quantification, cDNA was synthesized using QuantiMir Kit (System Biosciences) following the manufacturer's protocol. The universal reverse primer in the kit was used for all the reactions. Specific forward primers were used for miR-pgrn-1 (5′- TCGTTATTCTAGGCCATGTGTC-3′), miR-pgrn-9 (5′-TTGGATAGGCACTTACTCTGG-3′), miR-E1k (5′-TTGATGTGCAAGATCTTCTCAT-3′), miR-Scr (5′-TTGATAGAACCTTAGAGCATCG-3′) and mouse U6 (5′-TGGCCCCTGCGCAAGGATG-3′). The IQ Sybr Green mix (Bio-Rad) was used for the PCR reactions. The PCR was programmed as follows: 5 minutes at 95°C, followed by 40 cycles of 15 seconds at 95°C, 5 seconds at 56°C, and 5 seconds at 72°C. ΔCt values were calculated by subtracting the Ct value of the U6 from that of various miRNAs. ΔΔCt values were derived by subtracting the ΔCt of the wild type background level from the ΔCt of the single transgenic and double transgenic mice, which were then used to derive the fold increase of the miRNA over the wild type background level. All the qPCR reactions were done on the OPTICON system (MJ research).

### Histological analysis

Adult mice under deep anesthesia were transcardially perfused with cold PBS followed by cold fixation buffer containing 4% paraformaldehyde in PBS. Tissues were immersed in the same fixative overnight at 4°C. Mouse embryos were dip-fixed in the fixative overnight at 4°C. After fixation, tissues were immersed in PBS containing 15% sucrose for 2–3 days at 4°C. Tissues were then frozen in OCT freezing media (Sakura, Torrance, CA) and stored at −80°C. Frozen sections were cut at 10 µm using a cryostat and air-dried on slides at room temperature for 1 hour. For immunofluorescence staining, slides were incubated in the blocking solution (5% normal goat serum, 0.2% Triton X-100, 2% fat-free dry milk in PBS, pH 7.4) for 30 min at room temperature (RT), rinsed three times in PBS, and incubated with a primary antibody in the blocking solution overnight at 4°C. The slides were then rinsed with PBS three times and incubated with a secondary antibody in the blocking solution for 2 hrs at RT in dark. After washing, the slides were mounted with Vectashield mounting medium containing 4′,6′-diamidino-2-phenylindole (DAPI, Vector Laboratories). Images were taken using Nikon Eclipse Ti microscope equipped with NIS elements software and the three-dimensional deconvolution program Autoquant (Nikon). The antibodies used were RFP antibody (Clontech,#632496), human/mouse Caspase 3 active antibody (R&D systems, AF835), Alexa Fluor 568-conjugated goat anti-rabbit IgG (Invitrogen). For terminal deoxynucleotidyl transferase dUTP-X nick end labeling (TUNEL) to detect 3′-hydroxy termini of DNA strand breaks, frozen sections were prepared as stated above and proceeded using an In Situ Cell Death Detection kit (Roche) according to the manufacturer's instructions. Routine Hematoxylin and Eosin staining (H&E) was performed by Univeristy of Massachusetts Medical School DERC morphology core.

### Statistics

Statistical analysis of Mendelian ratio was done using Chi Square method (Table 2). Brain weight comparisons between the double transgenic and the wild type were done using Student's t-test (Table S1).

## Supporting Information

Table S1 The weights are in grams (Mean±SD). Values from the double transgenic mice were compared with the wild type mice using Student's t test. The numbers in parentheses indicate the number of animals.
(DOCX)

## References

[1] Lakso M, Sauer B, Mosinger B, Lee EJ, Manning RW (1992). Targeted oncogene activation by site-specific recombination in transgenic mice.. Proceedings of the National Academy of Sciences of the United States of America.

[2] Orban PC, Chui D, Marth JD (1992). Tissue- and site-specific DNA recombination in transgenic mice.. Proceedings of the National Academy of Sciences of the United States of America.

[3] Grieshammer U, Lewandoski M, Prevette D, Oppenheim RW, Martin GR (1998). Muscle-Specific Cell Ablation Conditional upon Cre-Mediated DNA Recombination in Transgenic Mice Leads to Massive Spinal and Cranial Motoneuron Loss.. Developmental Biology.

[4] Matsumura H, Hasuwa H, Inoue N, Ikawa M, Okabe M (2004). Lineage-specific cell disruption in living mice by Cre-mediated expression of diphtheria toxin A chain.. Biochemical and Biophysical Research Communications.

[5] Brockschnieder D, Lappe-Siefke C, Goebbels S, Boesl MR, Nave KA (2004). Cell depletion due to diphtheria toxin fragment A after Cre-mediated recombination.. Mol Cell Biol.

[6] Fritsch L, Martinez LA, Sekhri R, Naguibneva I, Gerard M (2004). Conditional gene knock-down by CRE-dependent short interfering RNAs.. EMBO Rep.

[7] Coumoul X, Shukla V, Li C, Wang R-H, Deng C-X (2005). Conditional knockdown of Fgfr2 in mice using Cre-LoxP induced RNA interference.. Nucl Acids Res.

[8] Boillee S, Yamanaka K, Lobsiger CS, Copeland NG, Jenkins NA (2006). Onset and progression in inherited ALS determined by motor neurons and microglia.. Science.

[9] Yamanaka K, Chun SJ, Boillee S, Fujimori-Tonou N, Yamashita H (2008). Astrocytes as determinants of disease progression in inherited amyotrophic lateral sclerosis.. Nat Neurosci.

[10] Lobsiger CS, Boillee S, McAlonis-Downes M, Khan AM, Feltri ML (2009). Schwann cells expressing dismutase active mutant SOD1 unexpectedly slow disease progression in ALS mice.. Proc Natl Acad Sci U S A.

[11] Wang L, Sharma K, Grisotti G, Roos RP (2009). The effect of mutant SOD1 dismutase activity on non-cell autonomous degeneration in familial amyotrophic lateral sclerosis.. Neurobiology of Disease.

[12] Zhong Z, Ilieva H, Hallagan L, Bell R, Singh I (2009). Activated protein C therapy slows ALS-like disease in mice by transcriptionally inhibiting SOD1 in motor neurons and microglia cells.. The Journal of Clinical Investigation.

[13] Huang ZJ, Yu W, Lovett C, Tonegawa S (2002). Cre/loxP recombination-activated neuronal markers in mouse neocortex and hippocampus.. Genesis.

[14] Livet J, Weissman TA, Kang H, Draft RW, Lu J (2007). Transgenic strategies for combinatorial expression of fluorescent proteins in the nervous system.. Nature.

[15] Qiu L, Wang H, Xia X, Zhou H, Xu Z (2008). A construct with fluorescent indicators for conditional expression of miRNA.. BMC Biotechnology.

[16] Gibson GE, Sheu KF, Blass JP, Baker A, Carlson KC (1988). Reduced activities of thiamine-dependent enzymes in the brains and peripheral tissues of patients with Alzheimer's disease.. Arch Neurol.

[17] Baker M, Mackenzie IR, Pickering-Brown SM, Gass J, Rademakers R (2006). Mutations in progranulin cause tau-negative frontotemporal dementia linked to chromosome 17.. Nature.

[18] Cruts M, Gijselinck I, van der Zee J, Engelborghs S, Wils H (2006). Null mutations in progranulin cause ubiquitin-positive frontotemporal dementia linked to chromosome 17q21.. Nature.

[19] Tronche F, Kellendonk C, Kretz O, Gass P, Anlag K (1999). Disruption of the glucocorticoid receptor gene in the nervous system results in reduced anxiety.. Nat Genet.

[20] Kayasuga Y, Chiba S, Suzuki M, Kikusui T, Matsuwaki T (2007). Alteration of behavioural phenotype in mice by targeted disruption of the progranulin gene.. Behav Brain Res.

[21] Yin F, Banerjee R, Thomas B, Zhou P, Qian L (2010). Exaggerated inflammation, impaired host defense, and neuropathology in progranulin-deficient mice.. J Exp Med.

[22] Yin F, Dumont M, Banerjee R, Ma Y, Li H (2010). Behavioral deficits and progressive neuropathology in progranulin-deficient mice: a mouse model of frontotemporal dementia.. Faseb J.

[23] Ahmed Z, Sheng H, Xu Y-f, Lin W-L, Innes AE (2010). Accelerated Lipofuscinosis and Ubiquitination in Granulin Knockout Mice Suggest a Role for Progranulin in Successful Aging.. Am J Pathol.

[24] Grimm D, Streetz KL, Jopling CL, Storm TA, Pandey K (2006). Fatality in mice due to oversaturation of cellular microRNA/short hairpin RNA pathways.. Nature.

[25] McBride JL, Boudreau RL, Harper SQ, Staber PD, Monteys AM (2008). Artificial miRNAs mitigate shRNA-mediated toxicity in the brain: Implications for the therapeutic development of RNAi.. Proceedings of the National Academy of Sciences.

[26] Lewandoski M, Martin GR (1997). Cre-mediated chromosome loss in mice.. Nat Genet.

[27] Gregoire D, Kmita M (2008). Recombination between inverted loxP sites is cytotoxic for proliferating cells and provides a simple tool for conditional cell ablation.. Proc Natl Acad Sci U S A.

[28] Gordon JW, Ruddle FH (1985). DNA-mediated genetic transformation of mouse embryos and bone marrow–a review.. Gene.

[29] Jaenisch R (1988). Transgenic animals.. Science.

[30] Palmiter RD, Brinster RL (1986). Germ-line transformation of mice.. Annu Rev Genet.

[31] Lobe CG, Koop KE, Kreppner W, Lomeli H, Gertsenstein M (1999). Z/AP, a double reporter for cre-mediated recombination.. Dev Biol.

[32] Novak A, Guo C, Yang W, Nagy A, Lobe CG (2000). Z/EG, a double reporter mouse line that expresses enhanced green fluorescent protein upon Cre-mediated excision.. Genesis.

[33] Otsuji T, Matsumura H, Suzuki T, Nakatsuji N, Tada T (2008). Rapid Induction of Large Chromosomal Deletions by a Cre/Inverted loxP System in Mouse ES Cell Hybrids.. Journal of Molecular Biology.

[34] Zhu Y, Kim Y-M, Li S, Zhuang Y (2010). Generation and Analysis of Partially Haploid Cells with Cre-mediated Chromosome Deletion in the Lymphoid System.. Journal of Biological Chemistry.

[35] Matsumura H, Tada M, Otsuji T, Yasuchika K, Nakatsuji N (2007). Targeted chromosome elimination from ES-somatic hybrid cells.. Nat Meth.

[36] Mills AA, Bradley A (2001). From mouse to man: generating megabase chromosome rearrangements.. Trends in Genetics.

[37] Leppanen P, Kholova I, Mahonen AJ, Airenne K, Koota S (2006). Short and long-term effects of hVEGF-A(165) in Cre-activated transgenic mice.. PLoS One.

[38] Liu J, Lobe CG (2007). Cre-conditional expression of constitutively active Notch1 in transgenic mice.. Genesis.

[39] Lo A, Fung M, Au CL, Chan T, Sauer B (2004). Transgenic Mice Over-expressing Endothelin-1 in Testis Transactivated by a Cre/loxP System Showed Decreased Testicular Capillary Blood Flow.. Transgenic Research.

[40] Kamei K, Ishikawa TO, Herschman HR (2006). Transgenic mouse for conditional, tissue-specific Cox-2 overexpression.. Genesis.

[41] Streicher JM, Kamei K, Ishikawa TO, Herschman H, Wang Y (2010). Compensatory hypertrophy induced by ventricular cardiomyocyte-specific COX-2 expression in mice.. J Mol Cell Cardiol.

