# Supplementary material for: A Non-Specific Effect Associated with Conditional Transgene Expression Based on Cre-loxP Strategy in Mice
Source: PLoS One. 2011 May 10;6(5):e18778. doi: 10.1371/journal.pone.0018778 (PMC3091857; doi:10.1371/journal.pone.0018778)
Supplement: Table S1 — The weights are in grams (Mean±SD). Values from the double transgenic mice were compared with the wild type mice using Student's t test. The numbers in parentheses indicate the number of animals. (DOCX) [file pone.0018778.s001.docx]

| **Table S1. Brain weights** | |  |  |
| --- | --- | --- | --- |
| Tg line | Dtg (n) | Wt (n) | P value |
| miR-PGRN9 | 0.19±0.015 (8) | 0.41±0.026 (16) | 1.20E-16 |
| miR-PGRN1 | 0.40±0.021 (10) | 0.42±0.038 (14) | 0.39 |
| miR-E1k32 | 0.23±0.021 (3) | 0.43±0.028 (22) | 1.82E-11 |
| miR-E1k15 | 0.41±0.024 (7) | 0.42±0.014 (17) | 0.46 |
| miR-Scr103 | 0.18±0.022 (11) | 0.46±0.016 (11) | 2.21E-19 |
| miR-Scr35 | 0.21±0.019 (13) | 0.41±0.025 (23) | 1.05E-23 |
| miR-Scr97 | 0.23±0.024 (8) | 0.42±0.021 (35) | 4.74E-25 |
| miR-Scr105 | 0.39±0.015 (6) | 0.41±0.023 (18) | 0.11 |
| miR-Scr109 | 0.41±0.015 (7) | 0.41±0.015 (9) | 0.3 |
| miR-Scr113 | 0.46±0.006 (6) | 0.42±0.041 (9) | 0.15 |

The weights are in grams (Mean±SD). Values from the double transgenic mice were compared with the wild type mice using Student’s t test. The numbers in parentheses indicate the number of animals.
